# Digital health interventions for women in frontline public service roles: A systematic review of effectiveness in reducing substance use

**DOI:** 10.1371/journal.pdig.0001154

**Published:** 2026-01-06

**Authors:** Grace Williamson, Toslima Khatun, Kate King, Amos Simms, Simon Dymond, Laura Goodwin, Ewan Carr, Nicola T. Fear, Dominic Murphy, Daniel Leightley

**Affiliations:** 1 School of Population Health Sciences, King’s College London, London, United Kingdom; 2 King’s Centre for Military Health Research, Department of Psychological Medicine, King’s College London, London, United Kingdom; 3 School of Global Studies, King’s College London, London, United Kingdom; 4 Academic Department of Military General Practice, Defence Medical Services, Birmingham, United Kingdom; 5 Academic Department of Military Mental Health, King’s College London, London, United Kingdom; 6 British Army, London, United Kingdom; 7 School of Psychology, Swansea University, Swansea, United Kingdom; 8 Department of Psychology, Reykjavík University, Reykjavík, Iceland; 9 Faculty of Health and Medicine, Lancaster University, Lancaster, United Kingdom; 10 Department of Biostatistics & Health Informatics, King’s College London, London, United Kingdom; 11 Combat Stress Centre for Applied Military Health Research, Leatherhead, United Kingdom; Iran University of Medical Sciences, IRAN, ISLAMIC REPUBLIC OF

## Abstract

Frontline occupations, including military, healthcare, and first responders, often include frequent exposure to traumatic events, increasing the risk of substance use disorders (SUDs). Research has shown that those in high-intensity occupations are at higher risk of developing SUDs compared to the general population. Women face unique experiences related to substance use, including greater functional impairment and barriers to treatment access. Yet, understanding of the effectiveness of digital health technologies in addressing substance use among women in frontline occupations is limited. This systematic review evaluates the effectiveness of digital health interventions in reducing substance use among women in frontline roles. Four databases (PsycINFO, Ovid MEDLINE, Embase, PsycArticles) were searched for English language full-text articles (2007–2024) that (1) evaluated a digital intervention designed to reduce substance use, (2) reported changes in substance use outcomes such as frequency, intensity or duration, using validated tools (3) included current or former frontline public service workers, and (4) included women as the primary target population or as a subgroup within the sample. 13 papers met inclusion criteria, focusing on eight distinct web and mobile-based interventions for alcohol, tobacco and illicit substances. Most studies (n = 11) reported substantial post-intervention reductions in alcohol and tobacco use, although results for PTSD symptoms, illicit drug use, and quality of life were mixed. This review highlights the potential of digital health interventions for reducing substance use but underscores significant gaps in research. The scarcity of studies focused on women, small and heterogeneous samples, and focus on veterans limits the generalisability to women in frontline roles. These gaps present a pressing challenge in understanding gender-specific digital intervention efficacy. Future research should prioritise larger, representative samples of women across diverse frontline occupations to drive the development of digital technologies tailored to the unique challenges faced by women in these roles.

## Author summary

Women in frontline public service roles, such as first responders and military personnel, are frequently exposed to trauma, placing them at heightened risk for substance use and adverse mental health outcomes. While digital health technologies, including mobile apps and web-based tools, are increasingly being developed to address these issues, their effectiveness for women remains unclear. In this review, we examined 13 studies on digital interventions targeting substance use, including alcohol, tobacco, and illicit drugs, among individuals currently or formerly in frontline occupations. The findings showed promise, with digital health technologies helping reduce alcohol and tobacco use. However, results for other substances and mental health outcomes, such as post-traumatic stress disorder and quality of life, were mixed. Importantly, few studies focused on women, and those that did often lacked diverse and representative samples. This lack of focus limits our understanding of how well these interventions work for women in such demanding roles. We highlight the urgent need for future research that addresses these gaps by prioritising larger, more inclusive studies. Tailoring digital tools to meet the unique challenges faced by women in frontline occupations can ensure these interventions are both effective and accessible, offering much-needed support in managing substance use.

## Introduction

Addictive substances, including legal but regulated substances like alcohol and tobacco, illicit drugs such as cocaine, and prescription opioids, have a high potential for abuse and are among the leading contributors to the global burden of disease, including substantial societal costs and reduced quality of life [1]. According to the World Health Organization (WHO), alcohol consumption is a major risk factor globally, with 2.6 million deaths caused by alcohol consumption in 2019 [2]. While men typically show higher overall prevalence rates of substance use disorders (SUDs) [3], women exhibit unique vulnerabilities/experiences in the aetiology, progression, comorbidities, and treatment of SUDs, which are shaped by a complex interplay of biological, genetic, environmental, and behavioural factors [4].

Neurobiological and clinical evidence support the concept of ‘telescoping’, whereby women experience heightened sensitivity to the reinforcing effects of addictive substances, contributing to a faster progression from initial use to dependence, including greater severity in cravings and withdrawal symptoms [5]. This phenomenon was first identified in relation to alcohol and has been replicated in subsequent research with other substances, including stimulants such as cocaine, methamphetamine, and nicotine, as well as opioids and cannabis [5]. For instance, a study of US armed forces personnel found that although men tended to drink alcohol more heavily, women displayed equal or higher rates of dependence symptoms and risk for alcohol-related problems [6]. Additionally, women tend to face greater functional impairment and more severe medical and psychiatric comorbidities compared to men with SUDs [7].

The epidemiology of substance use reveals notable gender differences [8]. Globally, men consume more alcohol and experience greater alcohol-related harms than women; however, evidence indicates that alcohol use, binge drinking, and drinking frequency are rising among women [9]. This trend has contributed to a narrowing gender gap in alcohol use and related harms, particularly among recently born cohorts [10]. Similarly, data from the United Nations Office on Drugs and Crime indicate that while men constitute most drug users, women use certain substances at rates comparable to men. Women represent more than 40% of users of amphetamine-type stimulants (ATS), as well as non-medical pharmaceutical stimulants, opioids, sedatives, and tranquilisers. However, there remains a pronounced gender gap in access to treatment. Despite nearly half of all past-year ATS users being women, only one in five individuals receiving treatment for ATS-related disorders is female [11].

Frontline occupations include professions that provide services to protect the lives and safety of others. They encompass armed forces personnel and first responders, including police officers, firefighters, and frontline medical staff. Individuals in these roles are likely to experience multiple exposures to traumatic events over their service [9]. Cumulative traumatic exposures increase the risk of these occupational groups for developing adverse mental health outcomes, including substance use, post-traumatic stress disorder (PTSD) [12], depression, sleep deprivation [13], and suicidal ideation [14]. Rates of hazardous alcohol use in these groups are substantially higher than in the general population. For example, in a UK study, 67% of male and 49% of female armed forces personnel were found to drink at hazardous levels, compared with 38% of men and 16% of women in the general population [15]. This elevated risk is further demonstrated in studies of other frontline workers. A study of 656 US firefighters found that 58% of career and 40% of volunteer firefighters reported recent heavy drinking or binge drinking [16]. Additionally, in a survey of 1,913 women firefighters, nearly 40% reported binge drinking in the past month, with heavy drinkers over 2.5 times more likely to report depression or PTSD symptoms, and 40% more likely to have sustained on-the-job injuries in the past year compared to their peers [17]. Another study found that amongst US armed forces personnel without a prior history of alcohol use disorder (AUD) or SUD before deployment, experiencing high levels of personal life stress during deployment was associated with an almost doubled risk of developing AUD/SUD within three months post-deployment, and three times the risk of developing chronic AUD/SUD at three- and nine-months post-deployment [18].

Beyond the high demands of frontline roles, women working in male-dominated environments, such as the military, police, and other frontline roles, face unique challenges as they navigate gendered expectations that compound the inherent stressors of their occupations. Beyond the high demands of their jobs, they often must navigate gendered barriers that shape their experiences in these settings [19]. A key issue in this discourse is the pervasive masculine bias embedded within the hierarchical structures of these organisations [20]. From constructionist and feminist perspectives, gender theorists argue that the culture in these fields often reinforces hyper-masculine ideals, including dominance, leadership, and the pursuit of power, which can contribute to harmful behaviours such as substance use and excessive drinking [21].

Existing research on female firefighters highlights that women face work segregation [19], discrimination [22], tokenism [20], and sexual harassment [23]. Similarly, within the military, a typically male-dominated environment, research, although limited in the UK, suggests that many women experience negative gender stereotyping and sexism [24]. A recent systematic review found that gender inequality, gender-based discrimination and hostile work environments were described as pervasive [25]. Women in the military report that they had to work twice as hard to prove themselves in a male-centric environment where they often felt overlooked and undervalued, leading to feelings of isolation and exclusion [26]. International studies corroborate these findings, showing that the pressure to conform to masculine military norms can result in emotional strain, with experiences differing based on military role and branch of service [24].

These compounded risks may make women in these professions particularly vulnerable to developing SUDs and underscore the need for interventions that account for both occupational stressors and gender-specific challenges. Women also face unique barriers when seeking treatment for SUDs, including both psychological and practical challenges. Stigma, discrimination, caregiving responsibilities, concerns about the potential involvement of child protection services after seeking help, and lack of treatment accessibility often deter women from accessing help, resulting in delayed treatment and more acute needs upon entry into care [27,28].

In response to these challenges, digital health technology, including mobile health (mHealth) smartphone apps, web-based interventions, and online counselling, offers promising accessible solutions for addressing substance use among women [17,29,30]. These technologies provide accessible, flexible, and scalable support, helping to overcome barriers like logistical constraints and stigma. A recent viewpoint emphasised the urgent need for feminist intersectionality in digital health to address the unique needs of women [31]. Digital health technologies have the potential to promote gender equality in healthcare by improving access to treatment, overcoming geographical limitations, empowering women to manage their health data, addressing the specific challenges faced by women in front-line service occupations, and alleviating pressure on healthcare systems. While current research shows mixed results regarding the efficacy of these interventions, with some studies demonstrating small to medium effects [28], they represent a critical step toward improving treatment accessibility for women with SUDs, particularly in high-stress, male-dominated professions.

The cumulative effect of trauma in high-stress occupations and the increased sensitivity to the reinforcing effects of addictive substances may place women at heightened risk for SUDs in these professions. Given the unique challenges women face in both the onset and treatment of SUDs, it is crucial to assess whether digital health technologies can provide effective and accessible solutions tailored to their needs. This systematic review aims to examine the effectiveness of digital health technology in reducing substance use among women in frontline service occupations.

## Methods

### Design

The systematic review was prospectively registered with PROSPERO (CRD42023459786) and conducted in accordance with Preferred Reporting Items for Systematic Reviews and Meta-Analyses (PRISMA) guidelines to ensure transparency and best practice [32].

### Search strategy

The search strategy was developed using the PICOS (Population, Intervention, Comparison, Outcome) framework. Four electronic databases (PsycINFO, Ovid MEDLINE, Embase, and PsycArticles) were searched concurrently via Ovid. These databases were selected as they cover psychological, medical, and interdisciplinary research addressing the review question. Records were also identified through searching reference lists of key papers.

### Eligibility criteria

Databases were searched for papers from January 2007 to July 2024. This criterion was applied to identify records following the release of the first-generation Apple smartphone in January 2007, marking the point at which websites also became accessible via smartphones. Search terms were grouped according to population (e.g., “wom?n or female”), occupation (e.g., “police or fire or military”), intervention (e.g., “interven* or therap*”), mode of delivery (e.g., “digital health or digital technolog*”) and clinical presentation including categories of substance use (e.g., “substance misuse* or substance-related disorder*”) (see Table 1 for full PICO search terms).

**Table 1:** PICO search terms.

PICO	Search terms
Population	Wom?n OR Female* AND Police OR Fire OR Military OR Officer* OR Prison OR Correction* OR Guard* OR (‘Search and Rescue’) OR Coast Guard* OR Paramedic* OR Nurs* OR Physician* OR Doctor* OR Emergency Respon* OR Veteran* OR Soldier* OR Armed Forces OR Army OR Navy OR Airforce OR “RAF” OR Marine* OR Reserve* OR Home Guard OR National Guard OR Front Line OR First Respon* OR Public Service OR Personnel
Intervention	Digital Health OR Digital Technolog* OR Mobile Phone OR Mobile Device OR Computer-Assisted OR Virtual OR Internet OR Web OR Online OR Remote Measurement Technolog* OR Text Messag* OR SMS OR Smartphone OR Device OR App* AND Interven* OR Therap*
Comparison	N/A
Outcome	((Substance misuse* OR Substance abuse* OR Dual-Diagnos* OR Drug* OR Substance-Related Disorder* OR Alcohol-Related Disorder* OR Cessation) AND (Categories of substances: Alcohol) OR Cannabis OR Marijuana OR Hash OR Heroin OR Opioid* OR Methamphetamine* OR Amphetamine* OR Cocaine OR Crack OR Solvent* OR Nicotine OR Vap* OR Hallucinogen* OR GHB or Ecstasy OR MDMA OR Ketamine)

Studies were eligible if they met the following criteria: (1) English language journal articles, (2) human participants, (3) non-review papers, (4) assessed the effectiveness of an intervention, (5) experimental or clinical designs, (6) digital health intervention was delivered (e.g., web or smartphone-based), (7) focused on reducing substance use, (8) current or former frontline public service occupation populations, (9) women as target population or included in sample, (10) empirical data, (11) not grey literature, and (12) non-duplicate studies.

Grey literature was excluded to ensure that included studies met consistent standards of quality, comparability, and rigorous peer review. Secondary analyses of previously published primary studies were included only if they reported additional relevant outcomes. Filters were applied to limit the results to English-language articles, full-text articles, and human subjects. Duplicate records were removed using database filters and manually during data screening. Due to a lack of studies focused exclusively on women identified during scoping searches, the remit included studies with mixed-gender samples.

### Study selection and data extraction

Scoping searches were conducted in October 2023 to refine search terms to increase the sensitivity of the search. Upon agreement of the terms, a final scoping search was run in October 2023 which identified 3748 records. The search was rerun prior to data screening in July 2024, yielding 3761 records.

Two independent reviewers (GW and TK) initially screened the titles and abstracts of all retrieved records to determine eligibility based on the predefined inclusion and exclusion criteria. For the full-text review, one reviewer (TK) independently assessed all eligible articles, while the second reviewer (GW) reviewed 30% of the full-text records. Data extraction from the included studies was conducted collaboratively by both reviewers (GW and TK). Any discrepancies were resolved at each stage of the selection process through discussion or consultation with a third reviewer, where consensus was reached (DL).

Key contextual, methodological, and statistical details from each record were extracted and stored in a spreadsheet for comparison, analysis, and synthesis. Extracted information included study characteristics, intervention characteristics, primary outcome data for substance use, and secondary outcome data, including PTSD symptoms and quality of life. Where possible, results for women-only sub-samples were extracted and reported. As recovery goals for individuals are a heterogeneous and non-linear process, the review was not limited to abstinence from substances as a measure of intervention efficacy. Empirical referents also included decreased duration, frequency, and intensity of substance use or specific validated measures such as score reduction on the Timeline Follow Back (TLFB) or Alcohol Use Disorders Identification Test (AUDIT).

A narrative synthesis was conducted to summarise and interpret findings across the included studies, due to the variation in study designs, populations, interventions, and outcome measures. Extracted data were first presented in tabular form. This allowed for a descriptive comparison across studies. Findings were then grouped thematically, with studies organised according to intervention characteristics, delivery format (mobile app or web-based), and primary substance targeted (alcohol, tobacco, or other drugs). The synthesis also paid specific attention to female representation within study samples, whether gender-disaggregated outcomes were reported, and the relevance of each study to frontline occupational contexts. Intervention outcomes were synthesised narratively, with emphasis on reductions in substance use from baseline to primary endpoint. Secondary outcomes such as PTSD symptoms, coping skills, and quality of life were also synthesised to capture broader intervention effects.

### Quality assessment

The Standard Quality Assessment Criteria for Evaluating Primary Research Papers from a Variety of Fields was used to assess the quality of studies included in the systematic review [33]. The checklist addressed the research question, study design, sampling technique, outcome measures, analytic measures, confounding, results, and conclusions. One independent reviewer (GW) assessed the quality of each included study, and a second reviewer (DL) assessed the quality of one third of the records. Studies were rated on 14 criteria (0: No, 1: Partial, 2: Yes). A summary score was calculated for each paper (sum of total score divided by total possible score), giving quality scores ranging from 0 (lowest) to 1 (highest), with higher values indicating higher quality. Kmet and colleagues [33] suggest utilising a minimum threshold score of 0.55 for inclusion of studies.

## Results

### Study characteristics

**Fig 1:**
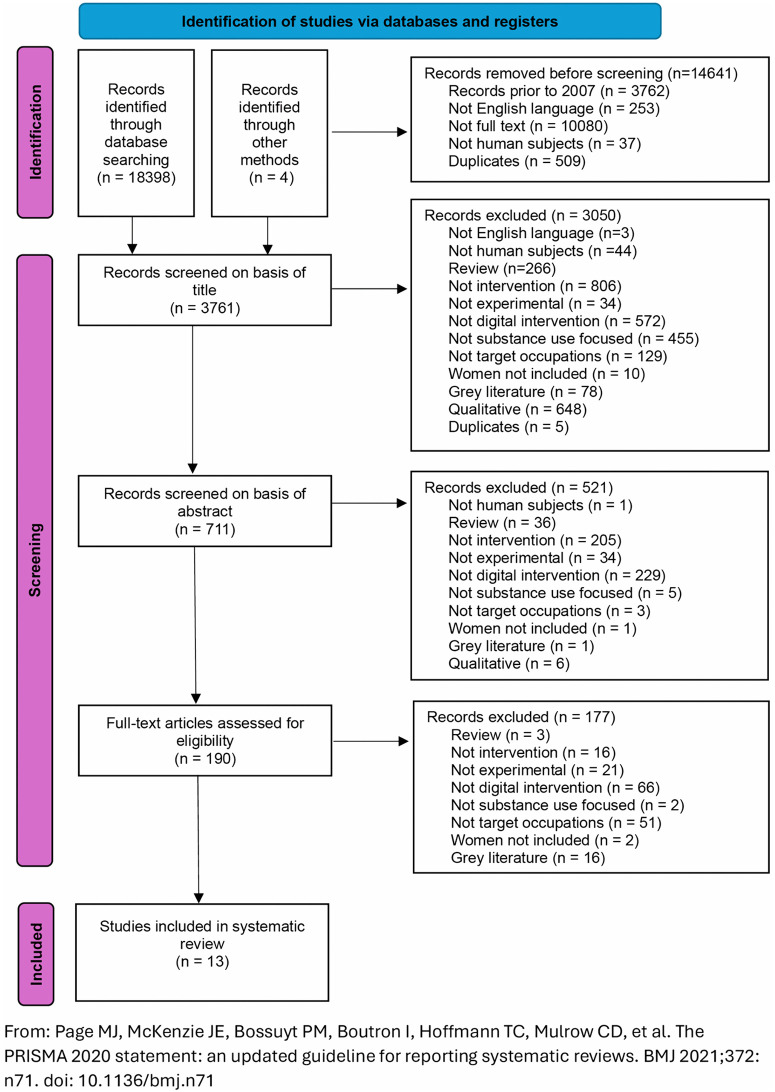
PRISMA flow diagram. From: Page MJ, McKenzie JE, Bossuyt PM, Boutron I, Hoffmann TC, Mulrow CD, et al. The PRISMA 2020 statement: an updated guideline for reporting systematic reviews. BMJ 2021;372: n71. https://doi.org/10.1136/bmj.n71.

A total of 18,398 articles were identified through the database search, with an additional four papers identified through other means, including searching reference lists of key papers (see Fig 1 for PRISMA flow diagram). After applying date, English language, human subjects, and full-text filters and removing duplicates, 14,641 articles were excluded. 3,761 titles and 711 abstracts were screened, with 190 articles identified for full-text screening. Of these, 177 were excluded as they did not meet the inclusion criteria.

At each screening stage, the interrater reliability between reviewers was calculated. The interrater reliability between reviewers for title screening was substantial (Cohen’s kappa (*κ*) of 0.72, an 89.23% agreement rate).

This review included 13 studies investigating the effect of digital health interventions aimed at reducing substance use among participants, with a focus on those in high-risk occupations (see Table 2 for full list of studies). The studies’ publication years spanned from 2013 to 2022 and were conducted primarily in North America (n = 12).

**Table 2:** List of individual studies.

Paper	Location	Study design	Sample size N	Female participants N (%)	Sex/gender-based analysis (Y/N)	Age in years Mean (SD)	Current or former occupation	Digital component	Intervention groups	Active intervention	Target substance
Acosta et al., 2016	North America	RCT	162	11 (7.0)	N	34.0 (8.1)	Veterans	Web-based	*Thinking Forward* CBT or TAU	24 self-paced modules including interactive exercises, veteran stories, CBT skills for managing PTSD symptoms and problematic substance use, and relaxation techniques and tools for insomnia and pain.	Alcohol and psychoactive substances (not specified)
Bell et al., 2017	North America	RCT	48	3 (6.7)	N	52.6 (8.6)	Veterans	Web-based	CRT + Work Therapy or Work Therapy	Five hours per week of CRT using auditory and visual Posit Science software, progressing from basic sensory tasks to complex memory exercises, alongside 15 hours of Work Therapy, with weekly group support sessions.	Alcohol, opiates, cocaine, or polysubstance
Blonigen et al., 2020	North America	Pre-post	31	2 (6.5)	N	54.6 (17.3)	Veterans	Mobile app	*Stand Down* + peer support	10 modules, including personalised feedback on drinking patterns, goal setting (moderation or abstinence), cravings management, relapse prevention strategies, and mood assessments. Users completed initial modules on drinking patterns and goals, followed by daily monitoring and weekly progress feedback toward their drinking goals.	Alcohol and psychoactive substances (not specified)
Brief et al., 2013	North America	RCT	600	82 (13.7)	Y	32.0 (7.8)	Veterans	Web-based	*VetChange* or delayed intervention	Eight self-guided modules included setting drinking goals, managing high-risk drinking situations, and developing coping strategies for internal triggers like stress and anger, incorporating motivational, cognitive-behavioural, and self-control strategies. Tailored feedback, home exercises, and self-monitoring supported ongoing progress toward drinking goals.	Alcohol
Brief et al., 2018	North America	Secondary analysis	523	72 (13.8)	N	31.9 (7.7)	Veterans	Web-based	*VetChange* or delayed intervention	Personalised feedback on alcohol use, alcohol-related problems, and PTSD symptoms. Participants monitored drinking, assessed readiness for change, set goals, and created a change plan with a support network. CBT strategies were introduced to help manage various high-risk drinking situations, such as social triggers and emotional states.	Alcohol
Enggasser et al., 2015	North America	Secondary analysis	305	40 (13.1)	N	31.7 (7.2)	Veterans	Web-based	*VetChange*	Key strategies included setting a personal drinking goal, self-monitoring, and completing assignments to support progress. Participants received guidance on selecting either an abstinence or moderation goal and were prompted to set a specific drinking target for the upcoming week.	Alcohol
Hicks et al., 2017	North America	Pilot RCT	11	7 (63.6)	N	53.8 (9.5)	Veterans & non-veterans	Mobile app	*QUIT4EVER Stay Quit Coach* or CCC	Personalised quit plans with tools to manage cravings, motivational messages, and relapse prevention support, and counsellors help participants customise the app during sessions, encouraging its use both between sessions and post-treatment.	Tobacco
Leightley et al., 2022	United Kingdom	RCT	123	6 (4.9)	N	47.6 (45.8-49.3)*	Veterans	Mobile app	*DrinksRation* or government guidance control	5 core modules based on BCTs including personalised insights based on drinking behaviour, self-monitoring and feedback with visual metrics for tracking alcohol intake, goal setting and review, and personalised messaging providing tailored reminders, alternative suggestions, and progress feedback through push notification.	Alcohol
Livingston et al., 2020	North America	Pre-post	222	50 (22.5)	N	36.0 (7.2)	Veterans	Web-based	*VetChange*	Self-guided modules included tools for mood and drink tracking, self-control training for high-risk situations, personalised feedback, motivational exercises, goal setting, building social support, and psychoeducation targeting PTSD-related challenges impacting alcohol use.	Alcohol
Miller et al., 2018	North America	RCT	571	97 (17.0)	Y	28.9 (3.3)	Veterans	Web-based	PNF or video game attentional control	Comparison of participant’s drinking habits (weekly drinks, drinks per occasion, and binge drinking days per month) to their perceptions of peers’ drinking patterns and drinking data from same-sex young adult veterans.	Alcohol
Pedersen et al., 2017	North America	RCT	396	70 (17.7)	Y	28.9 (3.4)	Veterans	Web-based	PNF or video game attentional control	Comparison of participant’s drinking habits (weekly drinks, drinks per occasion, and binge drinking days per month) to their perceptions of peers’ drinking patterns and drinking data from same-sex young adult veterans.	Alcohol
Possemato et al., 2019	North America	Pilot RCT	30	2 (7.0)	N	39.0 (9.0)	Veterans	Web-based	CBT *Thinking Forward* with or without peer support	24 modules including the connection between PTSD and substance use, motivational enhancement, relaxation techniques, identifying and challenging automatic thoughts, functional analyses of substance use, and substance use refusal skills. Interactive exercises and narratives from veterans illustrated common symptoms and healthy coping strategies. Participants were also assigned a peer support specialist for weekly meetings.	Alcohol
Willis et al., 2020	North America	Pre-post	117	90 (76.9)	N	37.38 (8.4)	Emergency dispatchers	Mobile app	*PTSD Coach*	Modules included deep breathing, mindfulness, muscle relaxation, and thought stopping, used whenever participants felt the need to manage stress.	Alcohol

BCT – Behavioural Change Techniques, CBT – Cognitive Behavioural Therapy, CCC - Combined Contact Control, CRT – Cognitive Remediation Therapy, PNF – Personalised Normative Feedback, PTSD – Post-traumatic Stress Disorder, RCT – Randomised Controlled Trial, TAU – Treatment as Usual.

*95% Confidence Interval.

### Population characteristics

Despite the focus of this review being on women in frontline public service roles, female representation was notably low in most studies, with the percentage of women below 25% in all but two studies [34,35]. Additionally, whilst the search strategy was designed to capture a wide range of current or former frontline occupations such as police, firefighters, military personnel, and healthcare workers, all of whom represent frontline or first responder occupations, most studies (n = 12) included in this review focused exclusively on veterans. Only one study specifically addressed a digital intervention for a different frontline occupational group, namely, emergency responders [35]. The sample sizes of studies varied considerably, ranging from smaller pilot studies [34,36] with sample sizes of n = 11 and n = 30, respectively, to larger randomised controlled trials (RCTs) with sample sizes of n = 571 and n = 600, respectively [37,38].

### Study designs

Most studies (8 out of 13; 61.6%) used randomised controlled trial (RCT) designs [30,34,36–41], often comparing the digital intervention to treatment-as-usual (TAU) or control conditions. Two studies were pilot RCTs [34,36], three studies utilised quasi-experimental pretest-post-test designs [35,42,43], and the remaining two studies were secondary analyses of an original RCT included in this review [44,45].

Some studies used longitudinal models to track alcohol use outcomes across multiple follow-up periods [43,44], while other studies utilised pre-post designs to measure the immediate and short-term impacts of the interventions [37,41].

### Target substances

Alcohol was the primary substance of interest in 12 of the 13 studies (92%) [30,35–45]. While some participants reported other substance use (e.g., cocaine, opiates, polysubstance use), these were typically not the primary targets of the interventions but rather reflected participant characteristics in two studies [39,42]. For instance, one study included various substances (n = 28 participants primarily used alcohol, n = 10 used cocaine, n = 6 used opiates, and n = 4 were polysubstance users with more than one primary drug in addition to alcohol) [40]. Only one study targeted tobacco as the primary substance [34].

Substance-related outcomes primarily focused on reductions in alcohol consumption, including frequency and quantity measures such as drinks per drinking day (DDD) and percent heavy drinking days (PHDD), alongside alcohol-related problems. Abstinence was less frequently the main outcome, consistent with harm-reduction approaches recognising diverse participant goals.

Secondary outcomes included PTSD symptoms [35,38,41,43,45] in the context of co-occurring PTSD and AUDs. Other outcomes across the studies included anxiety [35], depression, [35,41], readiness to change [39,42], self-efficacy [34,36,39] and usability of digital technology [30,34,35].

### Intervention characteristics

Interventions varied in format, delivery method, and duration. Most (n = 9; 70%) were web-based interventions [36–41,43–45] whilst four studies used mobile apps [30,34,35,42] to assess eight distinct interventions. The interventions were all multisession, and outcomes were assessed at various time points, including baseline and immediate post-intervention, with follow-up periods ranging from 1 to 6 months.

#### Cognitive behavioural interventions.

Of the 13 studies, four examined *VetChange* [38,43–45], a CBT intervention designed to help veterans reduce problematic drinking through motivational, cognitive-behavioural, and self-control strategies. Over eight modules, participants received personalised feedback on their drinking and PTSD symptoms, set drinking goals, and developed coping strategies for high-risk situations. The program emphasised self-management without the need for therapist involvement, with each module taking around 20 minutes to complete and including home exercises and self-monitoring to track progress and build coping skills.

Two studies [36,39] also used a CBT intervention - *Thinking Forward*. This self-directed program included 24 modules designed to teach cognitive-behavioural skills for managing PTSD and substance use, including interactive exercises, veteran stories, and strategies for managing symptoms like negative thoughts, insomnia, and trauma-related distress. One of these studies included peer support to examine whether engagement with the intervention and outcomes would be improved [36].

One study [42] used *Stand Down: Think Before You Drink,* a veteran-specific app designed for individuals aiming to reduce or abstain from alcohol use without engaging in in-person care. Grounded in motivational enhancement and cognitive-behavioural therapy, the app included 10 modules that addressed key areas such as assessing drinking patterns, setting goals (moderation or abstinence), managing cravings, and developing relapse prevention strategies.

#### Personalised normative feedback.

Two studies [37,41] adapted personalised normative feedback (PNF) interventions that compared participants’ drinking behaviours (e.g., weekly drinks, binge drinking) to their perceptions of and actual drinking patterns of same-sex young adult veterans.

#### Cognitive remediation therapy.

Bell and colleagues [40] used Cognitive Remediation Therapy (CRT) in which participants used auditory and visual Posit Science software, including the Brain Fitness (auditory) and Insight (visual) programs. Training began with simple sensory processing tasks and progressively became more complex, such as recalling details from audio stories. Participants also completed work therapy, a transitional work program, and attended group sessions for support concerning workplace issues.

#### Behavioural change theory.

One study [30] examined the efficacy of *DrinksRation*, an app designed to assist veterans who consume alcohol at hazardous or harmful levels, utilising behavioural change theory and personalised messaging. The app featured five core modules: account management, individualised normative guidance, self-monitoring, goal setting, and personalised messaging, allowing participants to track their consumption and receive tailored encouragement.

#### Psychoeducation and coping skills.

Willis and colleagues used a psychoeducation and coping skills app, *PTSD Coach,* to manage stress by engaging with various therapeutic modules such as deep breathing, mindfulness, and muscle relaxation [35]. Another study examined the *Stay Quit Coach* app, based on smoking cessation treatment tailored for individuals with chronic PTSD [34]. The app helps users who have quit smoking to maintain abstinence by creating personalised plans and providing tools to manage cravings, motivational messages, and support contacts. Counsellors assisted participants in customising the app during therapy sessions, and participants were encouraged to use it between sessions and after treatment to reinforce their progress.

### Quality assessment

Quality assessment scores were high with most papers (n = 11) reaching or exceeding 0.82, reflecting rigorous study design and implementation [30,35–38,40–45]. Only one paper scored significantly below this, with a summary score of 0.57 [34] (Table 3). All studies demonstrated clearly articulated research questions, appropriate and well-defined participant selection criteria, and the use of validated and reliable outcome and exposure measures. The predominance of RCTs contributed to the strength of the evidence base, with most studies (n = 12) employing suitable analytic methods and adequately reporting estimates of variance [30,35–45].

**Table 3:** Quality assessment of included papers.

Author, Year Published	Question/ objective (0–2)	Study design (0–2)	Subject/ comparison group selection (0–2)	Subject characteristics (0–2)	Random allocation (0–2)	Blinding of investigators (0–2)	Blinding of subjects (0–2)	Outcome and exposure measures (0–2)	Sample size (0–2)	Analytic methods (0–2)	Estimates of variance (0–2)	Controlled for confounding (0–2)	Results (0–2)	Conclusions (0–2)	Summary score (Mean)*
Acosta et al., 2016	2	2	2	2	2	0	0	2	1	2	2	1	1	2	0.75
Bell et al., 2017	2	2	2	2	2	2	2	2	0	2	2	1	2	2	0.89
Blonigen et al., 2020	2	2	2	2	N/A	N/A	N/A	2	1	2	2	0	2	2	0.86
Brief et al., 2013	2	2	2	2	2	1	2	2	2	2	2	1	2	2	0.92
Brief et al., 2018	2	2	2	2	1	0	0	2	2	2	2	2	2	2	0.82
Enggasser et al., 2015	2	2	2	2	N/A	N/A	N/A	2	2	2	2	N/A	2	2	1.00
Hicks et al., 2017	2	2	1	2	1	0	0	2	1	0	1	0	0	0	0.57
Leightley et al., 2022	2	2	2	2	2	2	2	2	2	2	2	2	2	2	1.00
Livingston et al., 2020	2	2	2	2	N/A	N/A	N/A	2	2	2	2	2	2	2	1.00
Miller et al., 2018	2	2	2	2	1	0	0	2	2	2	2	2	2	2	0.82
Pedersen et al., 2017	2	2	2	2	1	0	0	2	2	2	2	2	2	2	0.82
Possemato et al., 2019	2	2	2	2	2	0	0	2	1	2	2	2	2	2	0.82
Willis et al., 2020	2	2	2	2	N/A	N/A	N/A	2	2	2	2	N/A	2	2	1.00

2 – Meets criteria, 1 – Partially meets criteria, 0 – Does not meet criteria, N/A – Not applicable.

* A summary score was calculated for each paper (sum of total score divided by total possible score), giving quality scores ranging from 0 (lowest) to 1 (highest). Items not applicable to a certain study design were marked ‘N/A’ and excluded from the summary score calculation.

Despite these strengths, consistent limitations were observed in the domains of blinding and confounding control. Blinding of both investigators and participants was frequently absent or insufficiently described, which is a recognised challenge in psychological intervention trials in which blinding is often inadequately documented [46]. Moreover, a subset of studies (n = 5) did not fully account for potential confounding variables [34,38–40,42]. Sample sizes were generally adequate, although some studies (n = 5) were underpowered, limiting the precision and generalisability of their results [34,36,39,40,42]. The agreement rate between reviewers was 0.87, with discrepancies limited to one paper, indicating consistent and reliable application of the evaluation criteria across reviewers.

### Outcomes

Of the 13 studies evaluating the effectiveness of digital interventions for alcohol, substance use, and tobacco cessation, alcohol was the predominant target substance with most studies (n = 10) reporting significant reductions in consumption indicators such as DDD, PHDD, and alcohol withdrawal days (AWD) (see Table 4 for key findings). Common measures included the TLFB interview [30,36,39,40,42,44], Quick Drink Screen (QDS) [38,43–45], Alcohol Use Disorders Identification Test (AUDIT) [30,35,38,45], and the Daily Drinking Questionnaire (DDQ) [41]. Whilst some studies strengthened the reliability of subjective outcomes with collateral informants [39] or biological verification via breathalyser, urine toxicology [40], or CO testing [34], these approaches were not consistent across studies. Follow-up periods ranged from immediate post-intervention to six months, with limited evidence on longer-term outcomes.

**Table 4:** Summary of Outcome Measures and Key Results.

Paper	Study Groups	Primary Substance Use Measure(s)	Other Outcome Measure(s)	Key Findings
Acosta et al., 2016	*Thinking Forward* CBT or TAU	TLFB (PDD, PHDD, PDUD)	PCL-M WHOQOL-BREF	Significant reduction in PHDD for the intervention group vs. TAU (B = -1.80, SE = 0.79, p < .05). No significant intervention effects on PDD or PDUD.
Bell et al., 2017	CRT + Work Therapy or Work Therapy	TLFB, Breathalyser, urine toxicology screening	Battery of neurocognitive assessments	No significant difference in abstinence rates between groups at 3- and 6 months (87.3 vs 84.6 mean days abstinent, respectively).
Blonigen et al., 2020	*Stand Down* + peer support	TLFB (Total standard drinks, PDA, DPDD, PHDD), past month use (days) of substances identified by ASSIST	Interest in help for drinking, readiness to change alcohol use	Significant reductions in total drinks (~40%), PDA (~10%), DPDD (1.5 fewer), and PHDD (~15%). Non-significant trend for reduced rate of any drug use (42% to 24%, p = .06).
Brief et al., 2013	*VetChange* or delayed intervention	QDS (DPDD, AWD, PHDD)	PCL-5, SIP-2R	Significant reductions in DDD, AWD, PHDD, and PTSD symptoms in VetChange (baseline – end-of-intervention) vs. delayed intervention (baseline – end of waiting period (p < .001).
Brief et al., 2018	*VetChange* or delayed intervention	QDS (DPDD, AWD, PHDD)	PCL-5, SIP-2R	Participants with higher baseline PTSD showed significantly sharper declines in DDD (-0.004, p < .05), AWD (-0.007, p < .05), and PHDD (-0.002, p < .05) during intervention. At 3 months, high-PTSD participants averaged 3.6 DDD and 13% PHDD.
Enggasser et al., 2015	*VetChange*	QDS (DPDD, AWD, PHDD)	SIP-2R	Significant reductions in all alcohol outcomes across all goal-based groups (Abstinence Only, Abstinence to Moderation, Moderation to Abstinence and Moderation only). The Abstinence Only group showed the largest reductions (DDD: B = -.061, p < .001; AWD: B = -.100, p < .001).
Hicks et al., 2017	*QUIT4EVER Stay Quit Coach* or CCC	The Morisky Adherence Questionnaire, TLFB and smoking abstinence bioverification (expired air CO concentration or salivary cotinine)	N/A	High CO-verified abstinence rates post-treatment (Intervention: 60%, Control: 100%) and at 2-week follow-up (Intervention: 60%, Control: 67%).
Leightley et al., 2022	*DrinksRation* or government guidance control	TLFB, AUDIT	PHQ-2, GAD-2, ITQ	Intervention group had significantly greater reductions in weekly alcohol units (-15.4 units, p = .003, d = 0.35) and AUDIT scores (-3.9 points, p = .003, d = 0.48) at 84-day follow-up vs. control
Livingston et al., 2020	*VetChange*	QDS (AWD)	PCL-5	Significant reduction in alcohol withdrawal days (b = -0.61, p < .001) and weekly drinks from 39.4 (SD = 25.6) at baseline to 24.6 (SD = 22.6) at 1-month, with reductions maintained.
Miller et al., 2018	PNF or video game attentional control	DDQ (drinks per week, binge days), BYAACQ	PCL-5, PHQ-8	PNF was most effective for veterans with past-month alcohol-induced blackouts, who showed greater reductions in drinks/week (b = -6.41, p < .001) and consequences (b = -2.62, p < .001) than those without blackouts.
Pedersen et al., 2017	PNF or video game attentional control	DDQ (drinks per week, drinks per occasion, binge days), BYAACQ, DNRF	PCL-5, PHQ-8	PNF group reported significantly greater reductions than control at 1-month in drinks/week (-3.4, d = 0.25, p < .05), drinks/occasion (-0.4, d = 0.17, p < .05), binge days (-1.0, d = 0.18, p < .05), and consequences (-1.0, d = 0.17, p < .05).
Possemato et al., 2019	CBT *Thinking Forward* with or without peer support	TLFB (PDD, PHDD)	PCL-M, WHOQOL-BREF	No significant between-group differences in alcohol use outcomes (e.g., DDD: d = .13, 95% CI [-.59,.84]; PHDD: d = .17, 95% CI [-.55,.89]). Significant improvements in PTSD, quality of life, resiliency, and coping were observed with no differences between conditions.
Willis et al., 2020	*PTSD Coach*	AUDIT	DAR-5, GAD-7, PCL-5, PHQ-9	Significant reduction in mean AUDIT scores from baseline (M = 3.3, SD = 3.7) to post-intervention (M = 2.5, SD = 2.9, p = 0.007).

Abbreviations: ASSIST - Alcohol, Smoking, and Substance Involvement Screening Test, AUDIT – Alcohol Use Disorders Identification Test, AWD – Average Weekly Drinks, BYAACQ - The Brief Young Adult Alcohol Consequences Questionnaire, DAR-5, Dimensions of Anger Reactions, DDQ – Daily Drinking Questionnaire, DNRF – Drinking Norms Rating Form, DPDD - Drinks Per Drinking Day, GAD-7 – Generalised Anxiety Disorder Scale, ITQ – International Trauma Questionnaire, QDS – Quick Drink Screen, PCL-5 – PTSD Checklist for DSM-5, PCL-M - PTSD Checklist-Military, PDA - Percent Days Abstinent, PDD – Percent Drinking Days, PDUD – Percent Drug Use Days, PHDD – Percent Heavy Drinking Days, PHQ-8/9 – Patient Health Questionnaire, PTSD – Post-Traumatic Stress Disorder, SIP-2R – Short Inventory of Problems Revised, TLFB – Timeline Follow-Back, WHOQOL-BREF - World Health Organization Quality of Life – Brief.

CBT-based interventions were the most frequently evaluated alcohol-focused digital interventions in this review and largely reported significant improvements in alcohol use outcomes. For instance, Acosta and colleagues reported significant reductions in heavy drinking days in the *Thinking Forward* intervention group (CBT and PNF) compared to TAU, although no significant treatment-by-time effects were found for PTSD symptoms or quality of life [39]. Improvements in drinking were associated with gains in coping, social support, self-efficacy, and future hope. Similarly, Blonigen and colleagues also observed a 40% reduction in total drinks and a 15% decrease in heavy drinking days, alongside a non-significant decrease in drug use [42]. In addition, Brief and colleagues found reductions in DDD, AWD, PHDD, and PTSD symptoms [45], whilst a secondary analysis revealed that higher baseline PTSD scores were associated with greater reductions in alcohol use and related problems at three-month follow-up [38]. Livingston and colleagues reported a 43% decrease in alcohol use over six months, though alcohol reductions correlated with increased PTSD hyperarousal symptoms [43], whilst Engasser et al. reported the largest effects among participants aiming for moderation or abstinence [44].

PNF-only interventions also showed effectiveness in reducing alcohol use and alcohol-related harm. Pedersen and colleagues observed significant improvements in perceived norms and targeted drinking outcomes after one month, including drinks per week, average drinks per occasion, binge drinking days and alcohol related consequences [41]. Participants in the PNF group reported lower perceptions of peer drinking and reduced drinking levels and related consequences. Miller and colleagues found normative feedback associated with greater decreases in drinks per week, particularly for individuals with prior alcohol-induced blackouts [37].

Other interventions, including BCT [30] and psychoeducation [34] also produced significant effects. Leightley and colleagues found that intervention participants showed a significant decrease in weekly alcohol units (–28.2 units, *d* = 0.35, 95% CI [–36.9, –19.5], p = .003), measured by the 7-day TLFB at 84 days post-intervention [30]. Hicks and colleagues reported high rates of CO-verified tobacco abstinence immediately post-treatment, although prolonged abstinence rates at three and six months were mixed [34]. Willis and colleagues also noted significant alcohol reduction on the AUDIT over six weeks, though long-term effects were not assessed [35]. In contrast, Possemato and colleagues found significant improvements in psychological and social quality of life, resiliency, and coping, though these changes did not impact alcohol use [36].

Abstinence-focused outcomes were explicitly reported in only a subset of studies. Within the VetChange trial, Enggasser et al. [42] found that participants who selected abstinence as a goal achieved some of the largest reductions in DDD, AWD, and PHDD, as well as alcohol-related problems. In the tobacco cessation study, Hicks et al. [34] also demonstrated high rates of CO-verified abstinence immediately post-treatment; however, prolonged abstinence rates at three and six months were mixed. Conversely Bell and colleagues found no significant difference in abstinence rates between intervention and control groups at three and six months. However, overall abstinence rates were higher than those typically observed in comparable studies [40].

## Discussion

This systematic review found digital health interventions to consistently demonstrate significant reductions in alcohol consumption across key measures such as TLFB and AUDIT, supporting prior evidence of digital technology in addressing SUDs across various populations [47]. This highlights the promise of digital health interventions in reducing substance use, particularly alcohol, among individuals in frontline public service occupations.

Several interventions examined in this review, including *VetChange* and *Thinking Forward* [36,38,39,43–45], specifically targeted alcohol use among veterans. The finding that these programs produced significant reductions in alcohol use is consistent with the wider literature, which emphasises the role of CBT principles in promoting coping and emotional regulation skills, social support, and self-efficacy as mechanisms of change [48]. Digital platforms facilitate these processes by offering accessible, flexible, and stigma-reducing support, which may be particularly beneficial for populations with unique occupational stressors.

However, the differential effectiveness across outcomes warrants reflection. The significant reductions observed in alcohol use may be attributable to the targeted design of these interventions. Most programs were explicitly developed to address alcohol consumption using established techniques such as CBT, PNF, and self-monitoring, which directly engage with drinking behaviours, automatic cognitions, and triggers. In contrast, outcomes for illicit drug use, PTSD symptoms, and quality of life were mixed. This may be because these were often secondary outcomes in interventions primarily designed for alcohol reduction. For instance, while PTSD symptoms were frequently measured due to a high co-occurrence with AUD, the interventions may not have contained sufficiently intensive or specific trauma-focused components to reliably produce meaningful clinical improvements. Similarly, the lack of significant change in illicit drug use in the few studies that reported it [39,42] suggests that digital interventions that are effective for alcohol may not be directly transferable to other substances without adaptation to address distinct pharmacological and psychological drivers of use.

A critical limitation of the current evidence base is its overwhelming focus on veteran populations. This narrow focus means our review could not capture the potential effectiveness of digital interventions for the vast number of women in other frontline roles, such as police officers, firefighters, paramedics, and frontline healthcare workers. These occupations share similar risks of trauma exposure and substance use but operate within distinct organisational cultures and structural contexts. While many digital health tools demonstrate efficacy in general and veteran populations, the lack of tailored content informed by feminist and intersectional perspectives likely limits their impact for women in male-dominated, high-stress occupations. Theories discussed earlier, highlighting how gender norms, discrimination, and workplace culture contribute to substance use, imply that interventions neglecting these factors may not fully engage or support women. Incorporating feminist-informed approaches that address structural barriers, stigma, and caregiving responsibilities could enhance relevance, acceptability, and effectiveness.

The value of conducting gender-comparison analyses extends beyond simply determining if an intervention works “equally” for men and women. When performed in adequately powered studies, such analyses are essential for probing the equity of interventions [49]. They can reveal whether a one-size-fits-all approach is sufficient or if specific components resonate differently based on gender. For example, normative feedback (PNF) might be less effective for women if the reference norms are based on male drinking patterns. In this review, gender stratification was limited to two studies [37,41]. One study found that one month into the intervention, gender differences in alcohol-related outcomes showed trends suggesting that women may have reduced their drinking slightly more than men, though these findings were not statistically significant [37]. The authors note that although the study was adequately powered to detect a medium-sized gender effect, the small number of female participants restricted the ability to detect smaller effects. Another study concluded that the intervention’s effectiveness did not differ significantly by gender, suggesting that both men and women responded similarly to the intervention [41]. Collectively, these findings indicate that while current interventions may benefit both genders, additional research is necessary to determine how these tools might be adapted to better support women’s specific needs in addressing alcohol use within high-risk occupations, particularly as existing research shows that US and UK veterans, both male and female, report higher rates of heavy episodic drinking compared to civilian populations [50,51]. This gap is critical given the evidence that women in frontline roles face compounded risks, including biological sensitivity (‘telescoping’), occupational trauma, and gendered workplace stressors that may influence substance use patterns and treatment response differently than men.

Certain methodological limitations across the reviewed studies may have influenced the findings. Sample sizes varied widely, from small pilot studies (n = 11) [34] to large RCTs (n = 600) [38], potentially introducing bias where smaller studies may overestimate effect sizes. Most studies had short follow-up periods, limiting insights into long-term efficacy, a gap noted by Willis and colleagues, who observed immediate reductions in drinking but did not assess sustained effects, reporting this as a limitation of their study [35]. Furthermore, by excluding grey literature, potentially innovative approaches found in dissertations or unpublished studies were omitted, such as a recent dissertation on a mental health app for Canadian police officers designed to destigmatise and support substance use issues [52]. In addition, the evidence base is dominated by studies conducted in North America, particularly among US veteran and military populations. This geographical concentration may limit the generalisability of findings to other national and occupational contexts where cultural norms, healthcare systems, and organisational structures differ, underscoring the need for more diverse, internationally representative research.

### Implications and future directions

This review underscores the critical need for more studies tailored to women, highlighting a key gap in current research. These findings call for intersectional research that addresses the distinct needs of women in frontline occupations. Expanding digital health solutions to public sectors such as emergency services and the military can improve mental health outcomes and resilience on a larger scale, but must be informed by inclusive, gender-sensitive research.

Future research should address gaps identified in this review. Large-scale trials with greater representation of women are essential to assess the long-term, gender-specific impacts of digital interventions on substance use. Additionally, the scarcity of studies examining substance use among frontline personnel, such as police officers, firefighters, and healthcare workers, highlights an important gap in the current literature. Encouragingly, protocols and usability testing for digital tools in these populations are underway; for example, a tailored mHealth app for UK police officers adopts a holistic approach to physical and mental health, including alcohol use, signalling that research in this area is progressing [53]. Further investigation is also needed to assess the effectiveness of digital interventions on substances other than alcohol, as current evidence is sparse on opioid, stimulant, and other drug use. This is particularly crucial given the complex trauma profiles and comorbidities prevalent among frontline workers. The development of trauma-informed, feminist intersectional digital tools offers an opportunity to provide flexible, stigma-free support tailored to the lived realities of women in these demanding public service roles, ultimately working toward closing longstanding gaps in treatment access and outcomes.

In conclusion, whilst this review synthesises evidence illustrating that digital interventions can effectively reduce alcohol use in veteran populations, it also highlights a disparity between the known risks faced by women in frontline roles and the available research of effective interventions to support them. The almost exclusive focus on veterans, the paucity of women in samples, and lack of gender-based analysis identified in this review suggest that the current digital health landscape is not yet equipped to address the unique, intersectional challenges faced by women across a spectrum of frontline occupations in reducing substance use.

## Supporting information

S1 Checklist PRISMA 2020 Checklist. From: Page MJ, McKenzie JE, Bossuyt PM, Boutron I, Hoffmann TC, Mulrow CD, et al. The PRISMA 2020 statement: an updated guideline for reporting systematic reviews. BMJ 2021;372:n71. https://doi.org/10.1136/bmj.n71. This work is licensed under the Creative Commons Attribution 4.0 License (CC BY 4.0).
(DOCX)

S1 Data Screening and Data Extraction. (XLSX)

S1 File Search Terms. (DOCX)
