# Supplementary material for: Digital health interventions for women in frontline public service roles: A systematic review of effectiveness in reducing substance use
Source: PLOS Digit Health. 2026 Jan 6;5(1):e0001154. doi: 10.1371/journal.pdig.0001154 (PMC12773817; doi:10.1371/journal.pdig.0001154)
Supplement: S1 File — (DOCX) [file pdig.0001154.s003.docx]

S1 File. Search Terms

| 1 | Wom?n or Female* |
| --- | --- |
| 2 | Police or Fire or Military or Officer* or Prison or Correction* or Guard* or ('Search and Rescue') or Coast Guard* or Paramedic* or Nurs* or Physician* or Doctor* or Emergency Respon* or Veteran* or Soldier* or Armed Forces or Army or Navy or Airforce or “RAF” or Marine* or Reserve* or Home Guard or National Guard or Front Line or First Respon* or Public Service or Personnel |
| 3 | Digital Health or Digital Technolog* or Mobile Phone or Mobile Device or Computer-Assisted or Virtual or Internet or Web or Online or Remote Measurement Technolog* or Text Messag* or SMS or Smartphone or Device or App* |
| 4 | Interven* or Therap* |
| 5 | ((Substance misuse* or substance abuse* or Dual-Diagnos* or Drug* or Substance-Related Disorder* or Alcohol-Related Disorder* or Cessation) and Categories of substances: Alcohol) or Cannabis or Marijuana or Hash or Heroin or Opioid* or Methamphetamine* or Amphetamine* or Cocaine or Crack or Solvent* or Nicotine or Vap* or hallucinogen* or GHB or Ecstacy or MDMA or Ketamine) |
